# Etiopathogenic and Therapeutic Considerations in a Multiple Sclerosis Case with Acute Toxic Hepatitis

**DOI:** 10.3390/reports8020038

**Published:** 2025-03-26

**Authors:** Maria-Melania Dumitru-Martoiu, Simona Petrescu, Cristina Aura Panea

**Affiliations:** 1 Department of Neurology, Elias Emergency and University Hospital, 011461 Bucharest, Romania; 2 Department of Clinical Neuroscience, General Medicine, University of Medicine and Farmacy “Carol Davila”, 050474 Bucharest, Romania

**Keywords:** multiple sclerosis, disease modified treatments, liver injury, toxin, ashwagandha, herbal supplement

## Abstract

**Background and Clinical Significance**: In multiple sclerosis (MS), there are many therapeutic options, but most of the available drugs can cause drug-induced liver injury (DILI) after the first infusions. A wide group of other drugs may induce liver injury, from simple anti-pyretic medication like Acetaminophen to various dietary herb supplements like Ashwagandha. **Case Presentation**: A 39-year-old female patient, diagnosed with MS, has been previously treated with Glatiramer Acetate and interferon-beta, and is currently undergoing immunomodulatory treatment with natalizumab (infusion no. 81). She had a recent history of an airway infection for which she took 4–5 capsules of Acetaminophen per day for 7 days, along with the consumption of dietary supplement with Ashwagandha herb. She presented with jaundice, pruritus, and lower limb ecchymoses. The laboratory results revealed higher aminotransferase levels, total bilirubin, and alkaline phosphatase. The screening for autoimmune and infectious hepatitis was negative. The scenario of toxic hepatitis induced by recently used drugs (Ashwagandha dietary herb supplement and Acetaminophen) was adequate to start therapy with oral cortisone. The clinical and laboratory results gradually improved, with normal levels of liver enzymes and bilirubin, with no further increase after the discontinuation of corticosteroid therapy and dietary herb supplements. **Conclusions**: This case highlights the challenges in determining the multiple etiologies and managing acute liver injury in an MS patient on natalizumab, an immunomodulatory drug that can induce liver injury after the first infusions, especially in the context of recent ingestion of hepatotoxic drugs.

## 1. Introduction and Clinical Significance

Drug-induced liver injury (DILI) is a complex hepatic condition marked by abrupt hepatic damage caused due to exposure to hepatotoxic substances or their metabolites [1,2]. DILI can be categorized into two types: dose-dependent or intrinsic and dose-independent or idiosyncratic. Dose-dependent injury, such as seen in Acetaminophen overdose, can develop when the drug dose exceeds the limit for toxicity, with the extent of damage being proportional to the amount administered. On the other hand, dose-independent injury is not influenced by dose or treatment duration, frequently arising even at therapeutic doses [3].

A broad range of drugs may induce liver injury, from simple anti-pyretic drugs like Acetaminophen to multiple dietary herb supplements.

Acetaminophen is one of the most used analgesics, anti-pyretic, and anti-inflammatory drugs. While it is considered safe at therapeutic doses, overdoses of Acetaminophen can induce severe liver injury. After ingestion, 90% of the Acetaminophen is metabolized in nontoxic compounds (sulfate and glucuronide) in the liver and then excreted into the urine. Approximately 5–9% of Acetaminophen is metabolized by cytochrome p450 into a toxic form (*N*-acetyl-*p* benzoquinone imine—NAPQI) which can induce oxidative injury and hepatocellular necrosis. Only 2% of Acetaminophen is eliminated unchanged in the urine [4,5].

Dietary herb supplements become a major concern throughout the globe for liver injury [6]. Whitania Somnifera (or Indian Ginseng), popularly known as Ashwagandha, widely used in Indian Ayurvedic medicine [7], is a herb commonly used in supplements to improve stress and anxiety, enhance sleep and memory, support sexual function in both males and females, increase muscle mass and strength, and acts as “immune booster”. It can lead to liver injury, with the acute decompensation of cirrhosis, and acute-on-chronic liver failure. The primary pattern of liver injury is cholestatic, which may occur with or without inflammation and sometimes necrosis. Clinical presentation typically includes jaundice and pruritus [8].

In multiple sclerosis (MS), there are many therapeutic options, and most of the available disease-modifying therapies (DMTs) cause DILI.

DMTs, used to reduce the rate of relapses and accumulation of disability, can induce a group of immunological mechanisms, not being free of potential hepatic toxic effects [9,10]. The mechanism of liver injury during DMT’s treatment can be autoimmune, with viral reactivation, and in rare cases, with idiosyncratic reactions with unpredictable acute liver failure [2,9].

DMTs can be classified into moderate efficiency therapies like beta interferon, glatiramer acetate, teriflunomide, and dimethyl fumarate and highly efficient treatments like natalizumab, fingolimod, alemtuzumab, cladribine, and ocrelizumab, every therapy being individualized for every patient due to the risk–benefit profile, with induction and escalation strategies [11].

Natalizumab is a humanized monoclonal antibody anti-integrin that blocks the adhesion of leukocytes to vascular cell receptors on endothelial cells, thus preventing their migration into the central nervous system. Natalizumab was introduced for MS treatment for the first time in 2004, being administered in infusions at a dose of 300 mg and recently subcutaneously every 4 weeks [9].

Several of the adverse effects of natalizumab reported are rash, abdominal discomfort, nausea, depression, fatigue, urinary tract, and lower and upper respiratory tract infections. One important complication is progressive multifocal leukoencephalopathy, a neurological condition associated with John Cunningham virus reactivation in the central nervous system [9].

Liver injury induced by natalizumab can appear at any time during treatment. It has been reported after the first administration, or after multiple doses. There is an idiosyncratic autoimmune-like pattern, with hepatocellular involvement and sometimes with positive autoantibodies (anti-nuclear and anti-smooth muscle antibodies) [9,12].

Cases of liver injury or autoimmune hepatitis induced by natalizumab have been published in the literature, often presenting overlapping conditions, with autoantibodies and histological patterns of plasma cell infiltration. Notably, there had been no recurrence following the discontinuation of cortisone treatment [9].

## 2. Case Presentation

We present the case of a female patient diagnosed with MS, with natalizumab as immunomodulatory treatment initialized 7 years ago, who suddenly developed acute liver injury.

A 39-year-old female patient was diagnosed with MS at the age of 27 years old, with brainstem syndrome as disease onset. She experienced multiple relapses over the course of her condition and had undergone multiple therapies: (1) Glatiramer Acetate for one year and a half and (2) interferon-beta for two years. Both therapies were discontinued, due to treatment failure, with continued clinical and imaging activity. She was then escalated to current immunomodulatory treatment to (3) natalizumab (infusion no. 81), with normal liver function monitored monthly since the beginning of the treatment.

The patient had a recent history of an airway infection treated with 4–5 tablets of Acetaminophen per day for 7 days. She had a history of consumption of dietary supplements with Ashwagandha herb in the last month, with the desire to lose weight.

She presented at the emergency room with jaundice, pruritus, and lower limb ecchymoses. She had no fever, myalgia, diarrhea, or abdominal pain. She denied alcohol intake and use of illicit drugs.

On physical examination, she was overweight and there was no flapping tremor or other clinical signs of chronic liver disease. She was medically stable with a blood pressure of 110/60 mmHg, a pulse of 75 beats per minute, a respiration rate of 20 breaths per minute, and a normal temperature.

Abdominal ultrasound showed normal morphology of the liver, with no cholelithiasis or cholecystitis, and no dilatation of the biliary tract. An abdominal CT scan was conducted, showing no hepatic abnormalities.

The patient’s complete blood count showed a white blood cell (WBC) count of 11.9 × 10^3^/mL (segmented neutrophils 68.5%, lymphocytes 25%, eosinophil 0.3%), hemoglobin count of 12.3 g/dL, hematocrit count of 31.1%, and platelet count of 368,000/mL. The serum biochemical assay showed the following results: creatinine, 0.46 mg/dL; Na, 139 mEq/L; and K, 4.0 mEq/L. The laboratory results revealed aminotransferase levels higher than 1000 UI/L (ALT-2120 UI/L, AST—1208 UI/L), and higher total bilirubin (TB—7.9 mg/dL), direct bilirubin (DB—4.1 mg/dL), and alkaline phosphatase (ALP—247 UI/L). The changes in the hepatic enzymes reflected a mixed pattern of liver injury (hepatocellular and cholestatic), with a ratio of serum ALT to serum ALP of 4 (between 2 and 5) (Table 1).

In order to exclude the possible causes of acute liver injury, a screening for autoimmune and infectious hepatitis was performed.

The screening for autoimmune hepatitis was negative, with negative autoimmune autoantibodies (Ab): anti-nuclear Ab, anti-mitochondrial Ab, anti-smooth muscle Ab, and anti-liver kidney microsomal Ab (Table 2).

Screening for infectious hepatitis was negative. The anti-hepatitis A Virus Ab, Hepatitis B surface Antigen (Ag), anti-hepatitis B e-Ab and Ag, anti-hepatitis C Virus Ab, and anti-hepatitis E Virus Ab test results were all negative. There was no evidence of recent infection with Cytomegalovirus, Epstein–Barr Virus, or Herpes Simplex Virus (Table 3).

Overall, taking into account the clinical and paraclinical investigations, there was a scenario of mixed toxic hepatitis induced by recently used drugs (Acetaminophen and Ashwagandha herb dietary supplement). Treatment with oral prednisone at a dose of 40 mg/day was initiated.

The patient was followed up as an outpatient, with the corticosteroid dosage gradually reduced.

**Figure 1:**
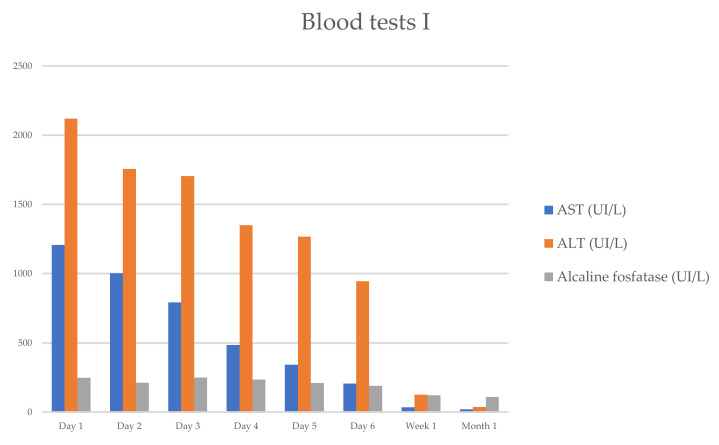
Evolution of serum biochemical assay (AST/ALT/ALP), which has markedly improved within 1 week.

**Figure 2:**
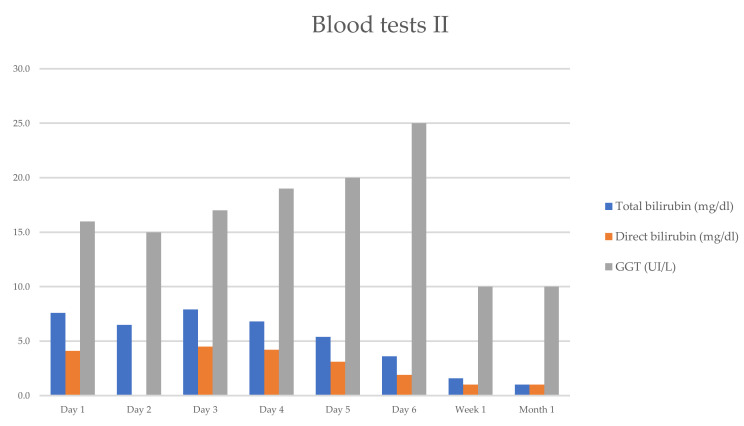
Evolution of serum biochemical assay (TB/DB/GGT), which has markedly improved within 1 week.

She progressively improved in clinical and laboratory test results Table 1 and Figure 1 and Figure 2, with the normalization of the serum levels of liver enzymes and bilirubin 1 month after starting the oral prednisone. The level of liver enzymes decreased after the discontinuation of corticosteroid therapy and dietary supplements.

Far from the normalization of the liver enzymes and from stopping the consumption of dietary herb supplements, the patient resumed the immunomodulatory treatment with natalizumab under 8 weeks after her last infusion, without changes in hepatic function in the following months (Table 1).

## 3. Discussion

We report the case of a female patient diagnosed with multiple sclerosis, currently receiving natalizumab (infusion no. 81) as immunomodulatory treatment. The patient developed drug-induced liver injury (DILI) related to Ashwagandha, which was precipitated by the use of the anti-pyretic drug Acetaminophen. The liver injury pattern was mixed—hepatocellular and cholestatic, with jaundice as the primary clinical presentation. A comprehensive diagnostic evaluation was performed to exclude other potential causes of the acute liver injury. Fortunately, the drug-induced liver injury was self-limiting and resolved with treatment and supportive care.

Natalizumab-induced liver injury is rare and may occur at any point during treatment, after the first or second infusion. Typically, it presents as mild acute hepatitis, which resolves spontaneously [9].

According to the phase III trials (AFFIRM, SENTINEL, and ASCEND), abnormal liver function tests were reported in 5% of patients receiving natalizumab as immunomodulatory treatment, with severe cases occurring in less than 1% of them. The data from these trials were collected after 2 years of treatment. However, extension studies up to 10 years confirmed that severe hepatobiliary adverse effects occurred in less than 1% of patients [13,14,15].

Since 2009, over 30 cases of patients with severe liver injury induced by natalizumab have been reported in the adverse event system, with an estimated incidence of liver injury from 17 to 100,000 exposed patients [9].

Ashwagandha offers many benefits, including increased exercise capacity, improved physical performance, better sleep, and reduced stress and anxiety.

Although generally considered safe, Ashwagandha has been associated with a high incidence of drug-induced liver injury. The mechanism of damage is believed to be related to the herb-specific compounds called withanolides, which can induce irreversible damage to hepatocellular DNA [16,17].

Reports of liver injury induced by Ashwagandha have been published in medical literature since 2011. The first case reports came from Japan, followed by multiple reports from the United States, India, Germany, and Poland. While the largest cohorts of Ashwagandha-related liver injuries have been reported, the actual number of cases may be higher.

Most herbal drugs contain multi-ingredient compounds, making it difficult to pinpoint the exact substance responsible for liver injury. In most cohorts, patients used only Ashwagandha supplements, establishing with certainty that Ashwagandha can cause severe liver injury and death in some cases [16,18,19,20,21,22,23,24]. In the case of our patient, she used only the Ashwagandha supplement.

Patients with chronic liver disease can develop more severe DILI, often resulting in a poor prognosis. Some reports have linked DILI in patients with a history of liver disease to a higher mortality rate, up to 16%, compared to almost 5% in patients without liver disease [16,18,19,20,21,22,23,24].

Our patient history had no history of chronic liver disease or alcohol use. However, she was taking the hepatotoxic drug Acetaminophen concomitantly.

In all the cohorts reporting Ashwagandha-related liver injury, comprehensive viral screening for hepatitis was performed, which was deemed highly unlikely to be the cause of the described liver injury mixed pattern [16,18,19,20,21,22,23,24]. Autoimmune hepatitis screening revealed positive autoantibodies in one cohort, but without the classical features of autoimmune hepatitis [16]. Mild elevations in autoimmune markers are commonly observed in patients with DILI, particularly in those with herb-induced liver injury [16].

Cases of liver injury or autoimmune hepatitis induced by natalizumab have been published in the literature, often presenting overlapping conditions, with autoantibodies and histological patterns of plasma cell infiltration. These cases showed no recurrence after cortisone withdrawal [9].

In our case, a thorough diagnostic was conducted to exclude other causes of the acute liver injury. The screening for viral and autoimmune hepatitis was negative, with no positive autoantibodies. The blood test results with changes in the hepatic enzymes reflected a mixed pattern of liver injury (hepatocellular and cholestatic).

In all the cohorts, the Ashwagandha supplement was used to reduce stress and anxiety in young or middle-aged people. Most patients consumed the herb according to the label instructions or as prescribed [16,18,19,20,21,22,23,24]. In our case, the patient used the Ashwagandha supplement as part of her diet program. The withdrawal of the supplement and treatment with immunosuppressive cortisone resolved the jaundice and the liver injury.

## 4. Conclusions

The case report highlights the challenges in establishing multiple etiologies and managing acute hepatitis in an MS patient receiving natalizumab as immunomodulatory treatment, a drug known to potentially induce liver injury, particularly after the first infusions, along with a recent history of ingesting hepatotoxic drugs.

The diagnosis of DILI is often one of the most challenging. A wide range of drugs can cause liver injury, including DMTs approved for MS treatment, which can have potential hepatotoxic effects, especially after the first administration. Simple anti-pyretic drugs like Acetaminophen and dietary herb supplements can induce liver injury. Ashwagandha, a herb extensively used in traditional Indian medicine, is a dietary supplement and a potential liver-injury factor.

Given the many documented cases of liver damage caused by Ashwagandha or other drugs, with unknown mechanisms behind the substances it contains, there should be high awareness among healthcare providers about patients using various drugs and herbs, who can present with symptoms and signs of liver injury.

Public education is essential to avoid the use of unrecommended and potentially harmful drugs and herb supplements, aiming to reduce the incidence of DILI.

## Figures and Tables

**Table 1:** Evolution of serum biochemical assay.

	Day 1	Day 2	Day 3	Day 4	Day 5	Day 6	Week 1	Month 1	Month 3	Month 6	Month 9
AST (UI/L)	1208	1003	793	485	343	207	35	20	19	20	25
ALT (UI/L)	2120	1757	1704	1350	1268	946	125	36	16	17	21
Total bilirubin (mg/dL)	7.6	6.5	7.9	6.8	5.4	3.6	1.6	1.2	1.2	1.1	1.0
Direct bilirubin (mg/dL)	4.1	4.0	4.5	4.2	3.1	1.9	0.9	1.0	0.9	0.9	0.8
Alkaline phosphatase (UI/L)	247	213	250	235	210	189	121	110	100	104	102
GGT (UI/L)	16	15	17	19	20	25	10	10	9.5	9	9.7

**Table 2:** Screening for autoimmune hepatitis.

Autoimmune Ab	Results	Normal Range
ANA	Negative	Negative
AMA M2	Negative	Negative
M2-3E	Negative	Negative
Sp100	Negative	Negative
PML	Negative	Negative
Gp210	Negative	Negative
LKM-1	Negative	Negative
LC-1	Negative	Negative
SLA/LP	Negative	Negative
SS-A	Negative	Negative
Scl-70	Negative	Negative
CENP A	Negative	Negative
CENP B	Negative	Negative
PGDH	Negative	Negative
ASMA	Negative	Negative
dsDNA Ab	0.1 UI/mL	Negative < 10.0

Abbreviations: ANA, Sp100, PML-antinuclear antibody, AMA M2, M2-3E, PGDH-anti-mitochondrial antibodies, GP210-anti-glycoprotein-210 antibody, LKM-1-anti Liver kidney microsome type 1 antibody, LC-1-anti-liver cytosol 1 antibody, SLA-LP-anti liver antigen/liver-pancreas antibody, SS-A-anti-Sjögren’s syndrome type A antibody, Scl-70-anti topoisomerase I antibody, CENP A/CENP B-anticentromere A/B antibody, ASMA-anti-smooth muscle antibody, dsDNA Ab-anti double stranded DNA antibody.

**Table 3:** Screening for infectious hepatitis.

	Results	Normal Range
CMV Ab IgG	68.4 UI/mL	Negative < 15.0
CMV Ab IgM	0.1	Negative < 1.0
HSV Ab IgM	<0.500	<1.0
Hepatitis A Ab IgM	Negative	<1.0
HBs Ab	1.7 mUI/L	<1.0
Hepatitis B HBe Ab	Negative	>1.10
Hepatitis B HBe Ag	Negative	<0.09
HEV Ab IgG	Negative	<0.56
HEV Ab IgM	Negative	Negative
EBV Ab IgG	77.8 UI/mL	<20
EBV Ab IgM	<10.0 UI/mL	<20

Abbreviations: CMV—cytomegalovirus, HSV—herpes simplex virus, HEV—hepatitis E virus, EBV—Epstein-Barr virus.

## Data Availability

The data used to support the findings of this study are included within the article.
